# Confined growth of ZIF-8 in dendritic mesoporous organosilica nanoparticles as bioregulators for enhanced mRNA delivery *in vivo*

**DOI:** 10.1093/nsr/nwaa268

**Published:** 2020-10-23

**Authors:** Yue Wang, Hao Song, Chao Liu, Ye Zhang, Yueqi Kong, Jie Tang, Yannan Yang, Chengzhong Yu

**Affiliations:** Australian Institute for Bioengineering and Nanotechnology, The University of Queensland, Brisbane, QLD 4072, Australia; Australian Institute for Bioengineering and Nanotechnology, The University of Queensland, Brisbane, QLD 4072, Australia; School of Chemistry and Molecular Engineering, East China Normal University, Shanghai 200241, China; School of Chemistry and Molecular Engineering, East China Normal University, Shanghai 200241, China; Australian Institute for Bioengineering and Nanotechnology, The University of Queensland, Brisbane, QLD 4072, Australia; Australian Institute for Bioengineering and Nanotechnology, The University of Queensland, Brisbane, QLD 4072, Australia; Australian Institute for Bioengineering and Nanotechnology, The University of Queensland, Brisbane, QLD 4072, Australia; Australian Institute for Bioengineering and Nanotechnology, The University of Queensland, Brisbane, QLD 4072, Australia; School of Chemistry and Molecular Engineering, East China Normal University, Shanghai 200241, China

**Keywords:** mesoporous organosilica nanoparticles, metal-organic framework, mRNA transfection, cellular delivery, bioregulator

## Abstract

Zeolitic imidazolate framework-8 (ZIF-8) and its composites have diverse applications. However, ZIF-8-based nanocomposites are mainly used as carriers in biomolecular delivery, with the functions of metal ions and ligands rarely used to modulate the biofunctions. In this work, dendritic mesoporous organosilica nanoparticles (DMONs) with tetrasulfide bond were used to confine ZIF-8 growth partially inside mesopores as a novel nanocomposite for mRNA delivery. Each component in the resultant DMONs-ZIF-8 contributed to mRNA delivery applications, including high loading benefitting from positively charged ZIF-8 and large mesopores of DMONs, endosomal escape promoted by the imidazole ring of ZIF-8, and long-term glutathione depletion mediated by both zinc ions and tetrasulfide bond. Combined together, DMONs-ZIF-8 demonstrated enhanced mRNA translation and better transfection efficiency than commercial products and toxic polymer-modified DMONs *in vitro* and *in vivo*.

## Teaser

Confined growth of ZIF-8 nanocrystals in the large mesopores of organosilica nanoparticles with tetrasulfide bond is reported to upregulate mRNA translation and enhance delivery performance.

## INTRODUCTION

As a classic type of metal-organic framework, the zeolitic imidazolate framework-8 (ZIF-8) has attracted much attention for a wide range of applications, e.g. energy storage [1], gas separation [2] and drug delivery [3]. Considering the small micropore sizes of ZIF-8, biomolecules are encapsulated by biomimetic mineralization of ZIF-8, including amino acids [4], proteins [5,6], plasmid [7,8] and CRISPR/Cas RNA [3], mainly embedded inside the particles or on the outer surface. Such a strategy is advantageous for enhanced *in vitro* delivery or improved protection of cargo molecules [7]. Nevertheless, ZIF-8 is mainly applied as a delivery vehicle, with the function of its chemical composition (specifically the metal ions, Zn^2+^) rarely used in regulating the protein translation in gene delivery applications. Recent research focus has been devoted to ZIF-8-based multifunctional composites, such as modification of ZIF-8 on various particles (e.g. MOF [9], metal [10] and mesoporous silica [11,12]). Notably, silica nanoparticles with controllable morphology, specifically dendritic mesoporous silica/organosilica nanoparticles with large pore sizes [13–16], have been widely applied as biomolecular delivery vehicles. Strategies have been developed to modify ZIF-8 on silica nanoparticles through surface functionalization with polyvinylpyrrolidone, carboxyl and imidazole groups [17]. However, current ZIF-8 modification is either conducted on solid spheres [18] or fully blocks the porous structure of silica nanoparticles [19]. Moreover, to our knowledge, the synthesis of composite ZIF-8/mesoporous silica nanoparticles (including organosilica nanoparticles) with large pore sizes for biomolecule delivery is rarely reported.

Advances in messenger RNA (mRNA) technology have sparked great interest in developing nanomaterial-based delivery systems to address limitations including instability and endosomal entrapment [20–23]. However, efficient delivery of mRNA to cells, especially antigen presenting cells (APCs, e.g. macrophages) is challenging [24]. To date, reported mRNA delivery strategies have mainly focused on control over physical interaction between mRNA and nanocarriers for improved mRNA transfection, with few taking the mRNA translation mechanism into consideration in nanomaterial design [25]. We revealed that suppressed mRNA translation was related to the relatively high glutathione (GSH) levels in APCs and demonstrated that tetrasulfide bond bridged dendritic organosilica nanoparticles (DMONs) modified by polyethylenimine (PEI) can upregulate mRNA translation in APCs [26]. The oxidation of GSH to oxidized GSH (GSSG) by tetrasulfide-induced dual biological functions, including activation of mammalian target of rapamycin complex 1 (mTORC1) for enhanced mRNA translation [27] and reduced glyceraldehyde 3-phosphate dehydrogenase (GAPDH) mediated translation inhibition [28]. However, as a result of intrinsic cellular metabolism, there was regeneration of GSH from GSSG catalyzed by glutathione reductase (GR) [29]. Thus, long-term GSH oxidation to GSSG would be of great importance to improve mRNA translation in APCs with intrinsically high GSH level. It has been reported that zinc ions mediate GR inhibition and reduce the GSSG reduction to GSH [30,31]. Besides, the imidazole group promoted endosomal escape may address PEI induced cytotoxicity [3,32]. Thus, it is hypothesized that ZIF-8 nanocrystals and DMONs composites could offer a new mRNA delivery platform specifically towards APCs.

**Scheme 1.**
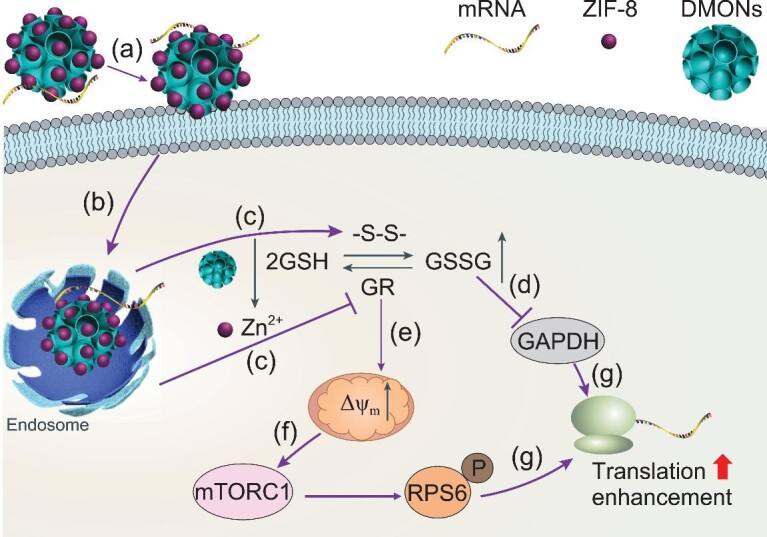
DMONs-ZIF-8 upregulate mRNA translation: (a) cellular uptake, (b) endosomal escape, (c) GSH depletion contributed by tetrasulfide-induced GSH oxidation and zinc-mediated inhibition of GR and GSSG reduction, (d) GAPDH deactivation, (e) increased MMP, (f) mTORC1 activation and (g) mRNA translation enhancement.

Herein, for the first time, we report synthesis of confined growth of ZIF-8 nanocrystals (∼23 nm) partially in the large mesopores (36 nm) of DMONs, taking advantage of the tetrasulfide bond embedded in the wall to adsorb zinc ions [33]. The resultant DMONs-ZIF-8 show advantages in mRNA delivery (Scheme 1), as a transfection agent for (a) high mRNA loading capacity enabled by large mesopores for cellular uptake; (b) successful endosomal escape contributed by acidic pH responsive breakage of zinc-ligand bonds in ZIF-8 [19]; and as a translation regulator for (c) synergistic GSH depletion by tetrasulfide-induced GSH oxidation and zinc-mediated inhibition of GR and GSSG reduction; (d–f) deactivated GAPDH involved mRNA translation inhibition and increased mitochondrial membrane potential (MMP) activated mTORC1 pathway; and consequently (g) enhanced mRNA translation and improved safety profile without using PEI modification. The prepared DMONs-ZIF-8 as bioregulators enable higher mRNA transfection efficacy compared with a polymer-modified control group (DMONs-PEI) as well as a commercial transfection agent (*in vitro*: lipofectamine, *in vivo*: *in vivo*-jetPEI). The understanding gained from this study provides new insights in the rational design of functional nanocarriers for mRNA delivery.

## RESULTS AND DISCUSSION

The DMONs were prepared according to a previous report (see details in Supporting data) [34]. Scanning electron microscope (SEM) (Fig. S1A) and transmission electron microscope (TEM) (Fig. S1B) images revealed the successful synthesis of DMONs with large pores. The cetyltrimethylammonium bromide (CTAB) removal was characterized by Fourier transform infrared spectroscopy (FTIR) analysis of DMONs before and after acid extraction (Fig. S2). Two characteristic peaks at 2855 and 2926 cm^−1^ originating from methylene groups of CTAB in as-synthesized DMONs disappeared in extracted DMONs, indicating the successful CTAB removal for DMONs after extraction. To modify ZIF-8 within the mesopores, DMONs were dispersed in a methanol solution of zinc nitrate at room temperature, then mixed with methanol solution containing 2-methylimidazole for ZIF-8 growth. After 36 h stirring, the final products were collected by centrifugation and washed with ethanol.

**Figure 1.**
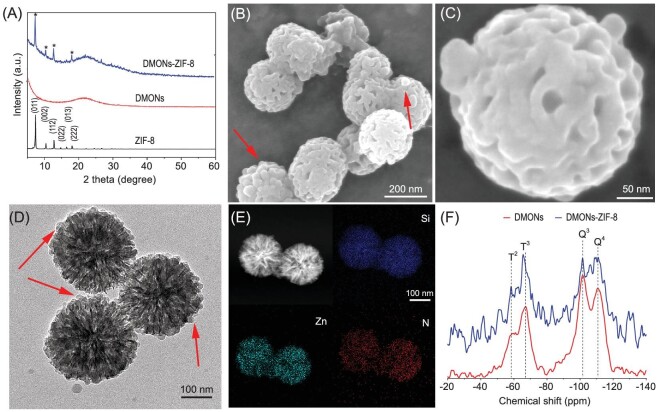
(A) XRD patterns of DMONs, ZIF-8 nanocube and DMONs-ZIF-8. (B and C) SEM images of DMONs-ZIF-8. (D) TEM image of DMONs-ZIF-8. EDX mapping images of DMONs-ZIF-8 (E) and ^29^Si solid state NMR spectrums (F) of DMONs and DMONs-ZIF-8.

X-ray diffraction (XRD) was conducted to characterize the crystalline structures. As a control sample, ZIF-8 nanocube with a uniform particle size of 188 nm (Fig. S3) was prepared according to a reported protocol [35]. The XRD pattern of ZIF-8 nanocube showed six peaks at 2θ range of 5 and 20°, which can be indexed to 011, 002, 112, 022, 013 and 222 diffractions (Fig. 1A), in agreement with the simulated pattern of sodalite ZIF-8 reported in the literature [36]. In contrast to the crystalline framework of ZIF-8, the XRD pattern of DMONs indicated an amorphous nature. DMONs-ZIF-8 exhibited typical peaks that can be indexed to 011, 002, 112, 013 and 222 diffraction of ZIF-8, although the 022 diffraction was not observed. Moreover, the diffraction peaks of DMONs-ZIF-8 were broadened. From the Scherrer equation, the crystalline domain of ZIF-8 was calculated to be 23.4 nm from the 011 diffraction with the highest peak intensity.

SEM images of the DMONs-ZIF-8 are shown in Fig. 1B and C. The deposition of some small nanoparticles on the external surface of DMONs can be observed, as indicated by arrows (Fig. 1B). Compared to the SEM image of DMONs with a large number of open pores, fewer open pores were observed on the external surface of DMONs-ZIF-8. TEM (Fig. 1D) was further conducted to characterize DMONs-ZIF-8. Compared to unmodified DMONs (Fig. S1), nanoparticles were found at the external surface of DMONs-ZIF-8 (indicated by arrows), leading to a relatively rough surface. This observation is consistent with SEM results. Moreover, the porous voids become less apparent compared to that of DMONs (Fig. S1B). Energy dispersive X-ray (EDX) elemental mapping (Fig. 1E) results indicate the overlapping of uniformly distributed Si, Zn and N elements in DMONs-ZIF-8. The above results collectively suggest the successful synthesis of DMONs-ZIF-8, where ZIF-8 nanoparticles were grown partially inside the large mesopores and extruded on the external surface. The average size of DMONs-ZIF-8 was calculated to be 235 nm by measuring ∼20 particles.

The pore structures were characterized by nitrogen sorption analysis (Fig. S4). Both DMONs and DMONs-ZIF-8 showed type IV isotherms and a major capillary condensation step at relative pressure (*P/P_0_*) of ∼0.8, suggesting the large pore of DMONs-ZIF-8 was maintained, while there was a decrease in pore size (from 36 to 34 nm) and pore volume (from 1.85 to 1.13 cm^3^/g) after ZIF-8 modification (Fig. S4B, Table S1), indicating the partial occupancy of ZIF-8 inside mesopores of DMONs. The surface area of DMONs-ZIF-8 was determined to be 483 m^2^g^−1^, notably higher than the surface area of 405 m^2^g^−1^ for pure DMONs. Elemental analysis (EA) showed a nitrogen content of 2.55% after ZIF-8 growth, while inductively coupled plasma optical emission spectrometry (ICP-OES) analysis revealed a Zn to Si weight ratio of 39% and a molar ratio of 16.7%. From the EA result, the weight ratio of ZIF-8/DMONs was estimated to be 10%. ZIF-8 has a low density of 0.95 g cm^−3^ and generally gives a specific surface area over 1000 m^2^g^−1^ [11,37]. The marginal increase in specific surface area of DMONs-ZIF-8 is possibly a result of the relatively low weight ratio of ZIF-8 inside DMONs.

The ^29^Si solid state NMR spectrums of both DMONs-ZIF-8 and DMONs are shown in Fig. 1F. The incorporation of a tetrasulfide motif in the framework of DMONs was evidenced by the existence of T^2^ (C-Si(OSi)_2_(OH)) and T^3^ (C-Si(OSi)_3_) species. A shift was observed in the characteristic peak of T^3^ in DMONs-ZIF-8 compared to that in DMONs, suggesting that Zn^2+^ was adsorbed on organosilica species. The ζ-potential of different samples in water was measured (Fig. S5). Compared to the positively charged ZIF-8 (+9.9 mV), the ζ-potential shifted from negative (−30.4 mV) for DMONs to positive (+3.2 mV) for DMONs-ZIF-8, also implying the successful growth of ZIF-8. The above results collectively support synthesis of ZIF-8 nanocrystals and tetrasulfide bond-bridged DMONs composites.

To understand the role of organosilica in the formation of DMONs-ZIF-8, inorganic dendritic mesoporous silica nanoparticles (DMSNs) prepared via calcination of DMONs were applied as a control. The ZIF-8 growth experiment was conducted under the same conditions for DMSNs. TEM images of DMSNs before (Fig. S6A) and after (Fig. S6B) ZIF-8 growth clearly exhibit a similar large dendritic mesopore channel. No zinc or nitrogen signals were observed from EDX mapping images after growth (Fig. S6C). The quantitative adsorption analyzed by ICP-OES was conducted by determining the Zn to Si weight ratio after DMONs or DMSNs were mixed with a zinc nitrate methanol solution, similar to the synthesis conditions. As shown in Fig. S7, DMONs exhibited a time-dependent increase in Zn/Si%, while no significant increase was observed for DMSNs, further indicating the higher adsorption of DMONs than DMSNs, consistent with the NMR result (Fig. 1F). Considering that (1) DMONs and DMSNs have similar structural properties; and (2) the ZIF-8 growth conditions are the same except for use of DMONs and DMSNs, it is inferred that the presence of tetrasulfide bond in DMONs is crucial for preparation of ZIF-8 nanocrystals and DMONs composites.

To investigate mRNA delivery performance, mRNA expressing enhanced green fluorescence protein (EGFP mRNA) was applied to investigate the mRNA loading capacity. PEI-modified DMONs (DMONs-PEI) (Fig. S8) with similar nitrogen content (2.21%) and ZIF-8 nanocube were selected as the control. As shown in Fig. S9, DMONs-ZIF-8 showed an EGFP mRNA loading capacity of 70 ng/μg, comparable to the loading capacity of DMONs-PEI (81 ng/μg), with both higher than the loading capacity of ZIF-8 (9 ng/μg). The slightly higher mRNA loading capacity of DMONs-PEI than that of DMONs-ZIF-8 is mainly explained by the relatively higher ζ-potential of 9.3 ± 0.9 mV than that of DMONs-ZIF-8 (+3.2 mV). The lowest loading capacity of ZIF-8 could be attributed to the absence of large mesopores compared to the other two samples. The mRNA loading content of DMONs-ZIF-8 was calculated to be 6.5 wt%, higher than ZIF-8 mediated biomimetic mineralization for plasmid (3.4 wt%) [7] or CRISPR RNA (1.2 wt%) [3] and electrostatic adsorption of siRNA (1.6 wt%) [38] as reported in the literature, highlighting the advantage of the large dendritic pore. These results reveal the significance of the maintained large pore structure of DMONs-ZIF-8 for high loading capacity and subsequent intracellular delivery of mRNA molecules compared to ZIF-8. The limited mRNA loading capacity indicated adsorption on the external surface of ZIF-8, thus ZIF-8 was excluded in the following mRNA delivery.

While ZIF-8 has been reported with biodegradability in phosphate buffered saline (PBS) to generate zinc phosphates [39], we further conducted FTIR and XRD analysis for DMONs-ZIF-8 after incubation in PBS (DMONs-ZIF-8-PBS) at 4°C for 30 min under the same condition for mRNA loading. The XRD pattern of DMONs-ZIF-8-PBS (Fig. S10A) exhibited typical peaks of ZIF-8 and no new diffraction peaks. As shown in the FTIR spectra (Fig. S10B), DMONs-ZIF-8-PBS exhibited typical peaks (1584 cm^−1^ and 1500–1350 cm^−1^) related to 2-methylimidazole and there was no obvious reduction in peak intensity compared to ZIF-8 and DMONs-ZIF-8 before PBS treatment. Moreover, no new vibration mode ascribed to zinc phosphate (1160–900 cm^−1^) was detected for DMONs-ZIF-8-PBS. These results all indicate no structural change of DMONs-ZIF-8 upon PBS exposure under the condition of mRNA loading.

To understand the effect of combined compositions on mRNA delivery efficiency, the RAW264.7 murine macrophage cell line was selected. The cytotoxicity assay showed that both DMONs-ZIF-8 and DMONs-PEI exhibited a dose-dependent cytotoxicity to macrophages; and DMONs-ZIF-8 exhibited higher cell viability than DMONs-PEI at all dosages tested. Both particles maintained more than 80% cell viability at concentrations up to 40 μg/mL (Fig. S11A). The good biocompatibility at this dosage is consistent with reports in the literature, where pure ZIF-8 exhibited no obvious toxicity up to 30 μg/mL [40]. This dosage was selected to mix with 1 μg of mRNA for the following *in vitro* mRNA delivery studies. We also evaluated the cytotoxicity of DMONs-ZIF-8 or DMONs-PEI towards HEK293T (human embryonic kidney) cells (Fig. S11B). Very limited cytotoxicity was observed for DMONs-ZIF-8 treated cells, with 75% of cell viability up to the dosage of 80 μg/mL, while relatively obvious dose-dependent cytotoxicity was shown in cells incubated with DMONs-PEI, with 42% of cell viability at 80 μg/mL. The cytotoxicity results reveal the good biocompatibility of DMONs-ZIF-8 over DMONs-PEI towards both immune cells and normal cells.

The ability of nanoparticles to deliver mRNA inside cells was investigated using EGFP mRNA labeled with Cy5 (Cy5-mRNA). RAW264.7 cells were incubated with Cy5-mRNA/nanoparticles or lipofectamine for 4 h. The relative amount of Cy5-mRNA inside cells was quantitatively analyzed by flow cytometry. As shown in Fig. S12A, the mean fluorescence intensity (MFI) of Cy5 in DMONs-ZIF-8/Cy5-mRNA was slightly higher than that in the lipofectamine group, but the difference is not significant. Both DMONs-ZIF-8 and lipofectamine groups showed significantly higher cellular delivery efficacy of mRNA in RAW264.7 cells compared to the DMONs-PEI group. Quantitative analysis of silicon uptake in RAW264.7 cells showed that the DMONs-ZIF-8 group had a higher cellular uptake (58 pg/cell of silicon) than the DMONs-PEI group (47 pg/cell, Fig. S12B), consistent with their intracellular mRNA delivery difference.

**Figure 2.**
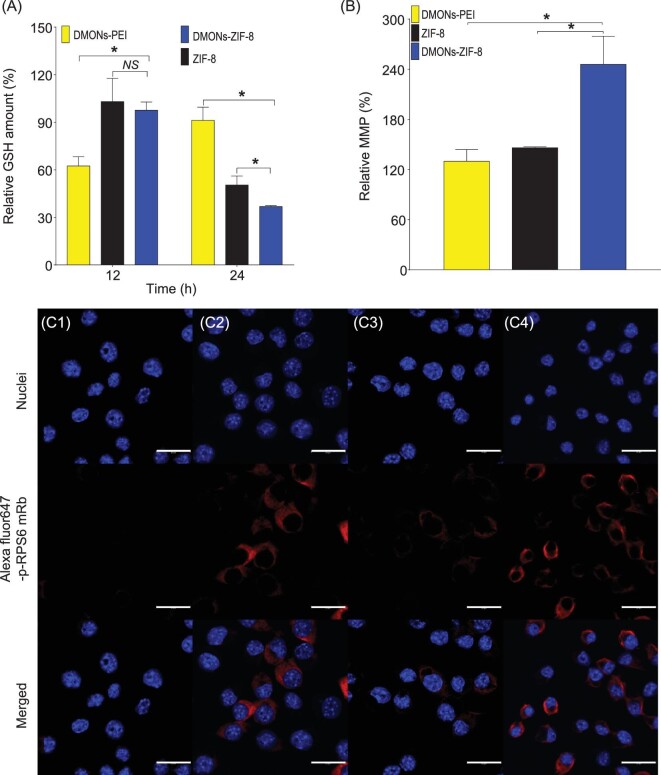
(A) Intracellular GSH level of RAW264.7 cells incubated with NPs for 12 and 24 h. (B) MMP of RAW264.7 cells incubated with NPs complex for 24 h. Immunostaining of Alexa Fluor 647 conjugated p-RPS6 antibody stained RAW264.7 after incubation with PBS (C1), ZIF-8 (C2), DMONs-PEI (C3) and DMONs-ZIF-8 (C4) for 24 h. Scale bars: 20 μm. Statistical analysis was performed using multiple t test. * shows *P* < 0.05 and *NS* indicates *P* > 0.05.

Next, the intracellular GSH amount was evaluated in RAW264.7 cells. ZIF-8 was added as the control, with the dosage for Zn content similar to that of DMONs-ZIF-8. As shown in Fig. 2A, a much lower GSH level was observed for DMONs-PEI than for ZIF-8 and DMONs-ZIF-8 at 12 h, which could be attributed to a redox reaction between tetrasulfide bond and GSH. At 24 h, there was a significant decrease in GSH level for cells treated with ZIF-8 and DMONs-ZIF-8 compared to 12 h, with both significantly lower than the DMONs-PEI treated group. The regenerated GSH in DMONs-PEI treated cells and continuous decrease in GSH level in ZIF-8 or DMONs-ZIF-8 treated cells indicated a significant role for ZIF-8 in prolonged GSH depletion.

To study the contribution of ZIF-8 to GSH depletion, GR activity was further examined. A significant reduction in GR activity (Fig. S13A) was shown for ZIF-8 (35%) and DMONs-ZIF-8 (34%), while the DMONs-PEI treated group displayed limited decrease (12%) in GR activity. It has been reported that zinc ions contribute to GR inhibition [30]. Thus, the lower GSH level contributed by ZIF-8 and DMONs-ZIF-8 (Fig. 2A) could be explained by the inhibited GR activity in the reduction from GSSG to GSH. The relative GAPDH activity was evaluated after incubation of RAW264.7 cells with different nanoparticles (Fig. S13B). The DMONs-ZIF-8 treated cells exhibited significantly lower GAPDH activity compared to DMONs-PEI (62% vs. 87%) and ZIF-8 treated cells (77%). The relative GAPDH activity supports our hypothesis that the tetrasulfide bonds of DMONs and zinc ions from ZIF-8 synergistically deplete GSH level and further deactivate GAPDH activity, beneficial for reducing GAPDH involved mRNA translation inhibition.

To investigate the advantage of ZIF-8 over zinc cations with DMONs, we also measured GR and GAPDH activity in RAW264.7 cells incubated with a mixture of DMONs and zinc nitrate at the same dosage as DMONs-ZIF-8. As shown in Fig. S13A, DMONs and zinc nitrate treated cells exhibited limited GR inhibition (18%), similar to the DMONs-PEI treated group and less than the DMONs-ZIF-8 treated group. The GAPDH activity (79%) was similar to that of DMONs-PEI and higher than that of DMONs-ZIF-8 treated cells (Fig. S13B). The limited GR and GAPDH inhibition in DMONs and zinc nitrate treated cells indicated the advantage of DMONs-ZIF-8 over other formulations where only Zn^2+^ was added with DMONs, supporting our hypothesis that both Zn and 2-methyl imidazole in ZIF-8 and tetrasulfide bonds in DMONs contribute to regulating intracellular biochemistry for enhanced mRNA delivery.

The MMP was evaluated by JC-1 staining and flow cytometry in RAW264.7 cells. Compared with control cells, DMONs-ZIF-8 treated cells (Fig. 2B) exhibited a significant increase in relative MMP among ZIF-8 or DMONs-PEI treated groups, responsible for the subsequent mTORC1 activation [41]. Activation of mTORC1 was studied by investigating the phosphorylation level of ribosomal protein S6 (RPS6) using immune staining analysis. The Alexa Fluor 647 conjugated p-RPS6 antibody was used to stain RAW264.7 cells after 24 h incubation with PBS or different nanoparticle formulations. Confocal microscope visualization demonstrated the highest phosphorylation level of p-RPS6 of DMONs-ZIF-8 treated cells (C4) (the strongest red signal) among PBS (C1), ZIF-8 (C2) and DMONs-PEI (C3) incubated cells, suggesting the highest mTORC1 activation level, beneficial for enhanced mRNA translation activity.

Next, the endosomal escape ability was visualized by confocal microscopy in RAW264.7 cells after incubation with DMONs-ZIF-8/Cy5-mRNA complexes (red fluorescence) for 8 h. The nucleus was stained with 4′,6-diamidino-2-phenylindole (DAPI) with blue fluorescence while the endosome was stained with lysosensor green with green fluorescence. DMONs-ZIF-8/Cy5-mRNA treated cells showed moderate yellow color (overlap of green and red colors from lysosensor green and Cy5-mRNA, respectively) in the cytosol, indicating limited entrapment of Cy5-mRNA in the endosome. The strong red color around the nucleus inside the cytosol (Fig. S14) suggested successful endosomal escape, which could be attributed to the proton sponge effect of the imidazole ring in ZIF-8 to promote endosomal escape [3]. After cellular uptake and entrapment in acidic endosome, ZIF-8 binds to protons and brings in counter ions, which increases ionic strength and osmotic pressure. The consequent water influx into endosomes causes endosome membrane rupture and released entrapped cargo molecules to achieve their functions.

**Figure 3.**
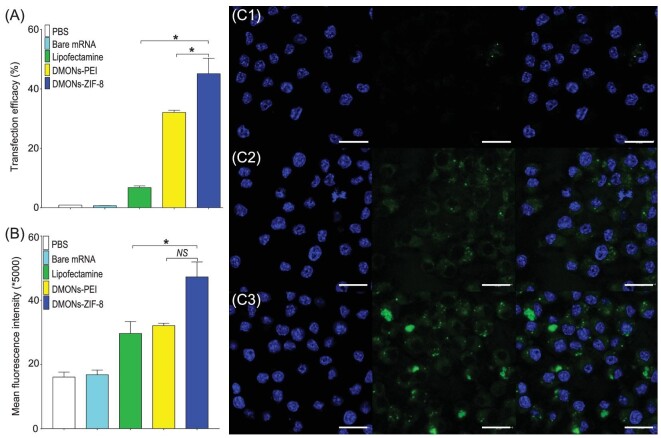
EGFP mRNA *in vitro* transfection in RAW 264.7 cells after treatment with mRNA-NPs for 48 h: flow cytometry analysis of positive cell percentage (A), mean fluorescence intensity (MFI) (B) and visualization by confocal microscope: lipofectamine-mRNA (C1), DMONs-PEI-mRNA (C2) and DMONs-ZIF-8-mRNA (C3). Scale bars: 20 μm.

Furthermore, flow cytometry was applied to evaluate EGFP expression after RAW264.7 cells were incubated with mRNA and three nanoparticle (NP) complexes for 48 h. Lipofectamine was also used as a control. DMONs-PEI/mRNA treated cells exhibited the highest transfection efficiency of 45.2% (Fig. 3A) compared to DMONs-PEI (32.0%) and lipofectamine (3.4%). The MFI of expressed EGFP is shown in Fig. 3B. The MFI of DMONs-ZIF-8/mRNA treated cells was significantly higher than that of DMONs-PEI and lipofectamine. The EGFP expression was also visualized by confocal microscopy. The nucleus was stained with DAPI with blue fluorescence. DMONs-PEI/mRNA treated cells showed the strongest green color (indicating the highest expression of EGFP) inside the cytosol compared to lipofectamine/mRNA (Fig. 3C1) and DMONs-PEI/mRNA (Fig. 3C2) groups. The high transfection efficacy of DMONs-ZIF-8 is speculated to be caused by synergistically upregulated mRNA translation of tetrasulfide bond-mediated GSH oxidation and ZIF-8-inhibited GR reduction of GSSG.

To evaluate the mRNA protection ability of nanoparticles against enzyme degradation, RNase was selected to investigate the impact of enzyme treatment on nanoparticles and lipofectamine-mediated mRNA transfection in RAW264.7 cells. Flow cytometry was used to quantitatively analyze the relative levels of EGFP mRNA transfection with RNase treatment compared to the same group without RNase. The EGFP-positive cell percentage remained similar (∼99%) in the RNase treated DMONs-ZIF-8 group compared to the untreated group (Fig. S15); however, a significant decrease was observed for DMONs-PEI (by 13%) and lipofectamine (by 83.5%) groups. A similar trend was observed in relative MFI change. The above results indicate that DMONs-ZIF-8 exhibit slightly better mRNA protection against RNase degradation compared to DMONs-PEI [17], and much better compared to the commercial transfection reagent, lipofectamine.

**Figure 4.**
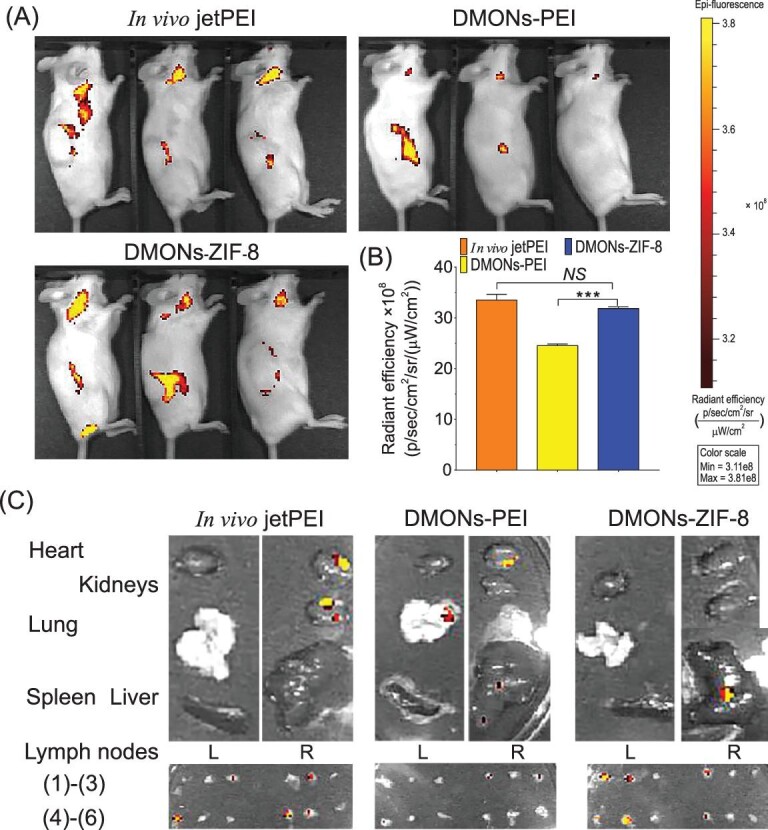
*In vivo* mCherry mRNA transfection in BALB/c mice. Fluorescence images of mCherry mRNA (A), quantitative analysis of fluorescence signals (B) and fluorescence signals (C) in major organs and lymph nodes ((1) and (2), superficial cervical; (3), brachial; (4), axillary; (5), inguinal; (6), sciatic. L, left side; R, right side) from *in vivo*-jetPEI, DMONs-PEI and DMONs-ZIF-8 injected mice.

Finally, we evaluated *in vivo* delivery of mCherry mRNA (with the same length to EGFP mRNA) in BALB/c mice to avoid autofluorescence interference [42]. Each formulation was subcutaneously injected at the lower back of BALB/c mice. After 48 h, the DMONs-ZIF-8/mRNA (Fig. 4A) treated group exhibited a stronger mCherry signal than the DMONs-PEI-mRNA complex, which is comparable to the commercial product, *in vivo*-jetPEI-mRNA injected mice. The mCherry signal of the DMONs-ZIF-8 group was observed to be mainly in superficial cervical, axillary and inguinal lymph nodes, while the signal in some mice of DMONs-PEI group is either undetectable or limited to particular lymph nodes. The quantitative result of average radiant efficiency at signal sites was calculated and is plotted in Fig. 4B, where the radiant efficiency was comparable between DMONs-ZIF-8 and *in vivo*-jetPEI treated mice. Major organs and lymph nodes of DMONs-ZIF-8 injected mice were collected to further evaluate the transfection efficiency (Fig. 4C). No obvious mCherry signal was detected for major organs while superficial cervical, axillary and inguinal lymph nodes exhibited strong fluorescence, consistent with the imaging results (Fig. 4A), suggesting efficient *in vivo* mRNA expression delivered by DMONs-ZIF-8 towards lymph nodes which are rich in immune cells.

The biodistribution of DMONs-PEI and DMONs-ZIF-8/mRNA formulations in major organs was monitored by measuring the residual silicon content (Fig. S16). Compared to the DMONs-PEI treated group, the DMONs-ZIF-8 treated group showed a lower silicon content in liver, but higher in lymph nodes, which is beneficial for accumulation of the mRNA formulation in lymph nodes for mRNA translation in immune cells. Long-term mCherry expression (Fig. S17) was observed for DMONs-ZIF-8 injected mice after 17 days while a very weak signal was detected for the commercial product, *in vivo*-jetPEI-mRNA transfected mouse. Our results have shown the potential for DMONs-ZIF-8 to replace the cytotoxic PEI used for mRNA delivery *in vivo*.

## CONCLUSION

In summary, ZIF-8 nanocrystals confined in the large mesopores of tetrasulfide bond bridged DMONs were prepared as bioregulators and applied to *in vivo* mRNA delivery for the first time. Both structure and composition of DMONs-ZIF-8 play important roles in successful mRNA delivery. The confined ZIF-8 growth with retained large pores is beneficial for high loading capacity and transfection efficacy of mRNA. The Zn^2+^, imidazole and tetrasulfide contribute to regulating the biofunctions associated with mRNA delivery, finally enabling both *in vitro* and *in vivo* transfection efficiency compared to commercial products and toxic polymer modification. The understanding drawn from this study paves the way towards the design of next-generation delivery vehicles for advanced mRNA applications.

## METHODS

The DMONs-ZIF-8 composites were prepared via a confined growth method. The comprehensive synthetic details, material characterizations and protocols for *in vitro* and *in vivo* studies are in the Supplementary data.

## Supplementary Material

nwaa268_Supplemental_File
