# Supplementary material for: Confined growth of ZIF-8 in dendritic mesoporous organosilica nanoparticles as bioregulators for enhanced mRNA delivery in vivo
Source: Natl Sci Rev. 2020 Oct 23;8(8):nwaa268. doi: 10.1093/nsr/nwaa268 (PMC8363327; doi:10.1093/nsr/nwaa268)
Supplement: nwaa268_Supplemental_File [file nwaa268_supplemental_file.docx]

Supporting Information

Confined ZIF-8 grown in large mesopores of organosilica nanoparticles as translation regulators for enhanced mRNA delivery

Yue Wang, Hao Song, Chao Liu, Ye Zhang, Yueqi Kong, Jie Tang, Yannan Yang and Chengzhong Yu*

Australian Institute for Bioengineering and Nanotechnology, The University of Queensland, Brisbane, QLD 4072, Australia

Materials and reagents

Triethanolamine (TEA), cetyltrimethylammonium bromide (CTAB), tetraethyl orthosilicate (TEOS, 98%), 1,4-bis(triethoxysilyl)-propane tetrasulfide (BTES), sodium salicylate, zinc nitrate, 2-methylimidazole, sodium 3-(trihydroxysilyl) propylmethylphosphonate (THPMP), polyethylenimine (PEI, branched, molecular weight: 10000), methanol (bioreagent, >=99.93%), phosphate buffer (PBS, 10Mm, pH7.4) and fetal bovine serum (FBS) were purchased from Sigma-Aldrich. mRNA expressing EGFP or mCherry was purchased from Trilink Biotechnologies. Lipofectamine 2000 and KDalert GAPDH assay kit were purchased from Thermo Fisher Scientific. In vivo-jetPEI was purchased from Polyplus Transfection. Intracellular glutathione (GSH) assay kit and alexa fluor 647 conjugated phosphor-S6 ribosomal protein (p-RPS6) antibody were purchased from Cell Signaling Technology. Glutathione reductase (GR) assay kit was purchased from abcam. Dulbecco’s modified eagle medium (DMEM) was purchased from Life Technologies. RAW264.7 marine macrophage cells and HEK293T human embryonic kidney cells were purchased from American Type Culture Collection (ATCC).

Synthesis of DMONs

In a typical synthesis, 190 mg of CTAB and 84 mg of sodium salicylate were added into 12.5 mL of deionized water containing 34 mg of TEA and stirred at 80 °C. After stirring for 1h, a mixture of 1.1 mL of TEOS and 0.7 mL BTES was added for further strirring for 12 h. Final product was collected by centrifugation and acid extraction to remove the surfactant. The final products are denoted as DMONs.

Synthesis of DMONs-ZIF-8 or DMONs-PEI

0.5 mg of DMONs were dispersed in 1 mL methanol solution by sonication and mixed with 5 mL of methanol solution containing 5.885 mg zinc nitrate, then vortexed with 5 mL of methanol solution containing 12.98 mg 2-methylimidazole for 10 s. After 36 h stirring at room temperature, DMONs-ZIF-8 were collected by centrifugation, thoroughly washed with ethanol and dried in oven at room temperature overnight.

The PEI modification was conducted by dispersing 30 mg of DMONs in 10 mL water (pH=10, adjusted by ammonia solution (25% assay)) and mixed with 10 mL of 56 mM THPMP solution and stirred at 40 °C for 2 h. Then samples were centrifuged, washed with ethanol twice and resuspended in 15 mL of 100 mM pH=9.6 carbonate buffer solution containing 150 mg 10K PEI stirring at room temperature for another 2 h. The final products were collected, centrifuged by washing with water and dried in a oven at 50 °C overnight.

Characterization

Transmission electron microscopy (TEM) images were taken using JEOL 1010 operated at 100 kV. Samples were firstly dispersed in ethanol and then dried on carbon film on a copper grid. The supernatant was deposited by droplets directly on the carbon film of a copper grid. Nitrogen adsorption-desorption measurement was conducted by a Micromeritcs Tristar II system at 77 K. Before measurement, samples were degassed at 373 K for 12 hours on a vacuum line. The total pore volume was calculated based on the adsorbed amount at the maximum relative pressure (P/P_0_) of 0.99. The pore size of samples was calculated through Barrrett-Joyner-Halenda (BJH) method from the adsorption branches of the isotherms. The Brunauer-Emmett-Teller (BET) method was used to calculate the specific surface areas. 29Si NMR were measured by the solid state Bruker Advance III spectrometer. Zeta potential was analyzed by dispersing samples in water and analyzed using the Zetasizer Nano-ZS (Malvern Instruments) at room temperature.

mRNA loading

10 μL of 0.5 mg/mL DMONs-ZIF-8, DMONs-PEI or ZIF-8 was mixed with 1 μL of mRNA solution (1 μg/μL) at 4 °C for 30 min. Then the mixture was centrifuged at 15,000 rpm for 10 min at 4 °C. The collected supernatant was analyzed by ‘Nucleic Acid’ application module at wavelength of 260 nm in a Nanodrop 1000 spectrophotometer (Thermo Scientific) to determine the residual concentration of mRNA, using PBS as the blank. The adsorbed amount of mRNA was calculated by the difference between original and residual concentration of the supernatant.

Cell viability assay

The cell viability was evaluated in RAW264.7 and HEK293T cells using MTT ((3-(4,5-Dimethylthiazol-2-yl)-2,5-diphenyltetrazolium bromide) assay. Cells were seeded in 96-well plates with a density of 12000 cells per well for 24 h. The cell culture medium was then replaced with fresh DMEM medium containing PBS or various concentrations of nanoparticles and incubated in a 37 °C, 5 % CO_2_ incubator. After 24 h incubation, 20 μL of 5 mg/ml MTT solution was added to each well for 4 h incubation. Then the medium was removed and 200 μL of DMSO was added to each well to measure the absorbance at the wavelength of 540 nm by the microplate reader using cells treated with PBS group as the control. All experiments were performed in triplicate.

Cellular uptake study

The cellular uptake DMONs-ZIF-8-mRNA or DMONs-PEI-mRNA complex in RAW264.7 cells was determined by inductively coupled plasms optical emission spectrometer (ICP-OES). Cells were seeded on the 12 well plate with the density of 1.2 ×10^5^ cells per well. After 24 h incubation, 50 μL of PBS solution containing 1 μg mRNA and 40 μg nanoparticles premixed for 30 mins was added to each well for another 4 h incubation. Then cells were washed with warm PBS and collected by washing with cold PBS. The cell number was counted and recorded for each sample. The cells were centrifuged and 120 μL of water added to each sample. After 1 h sonication, samples were centrifuged and precipitates were dried in the oven at 50 °C overnight. Then 150 μL of 1M NaOH solution was added to each sample for ICP-OES analysis.

Intracellular GSH, GR, GAPDH and p-RPS6

To determine the GSH, GR and GAPDH level, cells were seeded in the 96 well plate with cell density of 1.2 ×10^4^ cells per well. After 24 h incubation, DMONs-ZIF-8 or DMONs-PEI were added to each well at the concentration of 40 μg/mL while ZIF-8 were added at the final concentration of 4 μg/mL for similar zinc content to DMONs-ZIF-8. The GSH, GR and GAPDH level were determined according to the manufacture’s protocol at selected time points. The p-RPS6 level was analyzed by immunostaining assay. RAW264.7 cells were grown on sterilized cover slips inside 12 well plate with the cell density of 1.2 ×10^5^/mL and incubated for 24 h. Then same amount of nanoparticles were added to each well as GSH, GR and GAPDH studies and incubated for 24 h. The coverslips were washed with PBS, blocked with blocking buffer, stained in antibody at 4 °C overnight, washed with PBS again and stained with DAPI for nucleus staining for following visualization by confocal microscopy (Leica SP8).

*In vitro* mRNA transfection

RAW264.7 cells were grown inside the 12 well plate with a cell density of 1.2 ×10^5^/mL for 24 h incubation. Then 50 μL PBS solution of premixed mRNA and nanoparticles (1 μg mRNA and 40 μg nanoparticles) was added by droplets to each well for another 48 h incubation. Cells were collected and analyzed by flow cytometry or confocal microscope to determine the EGFP expression.

To demonstrate the protection capability of nanoparticle formulations, 0.05 mU of RNase was added into the mixture of nanoparticles/lipofectamine-mRNA and incubated at 37 °C for 30 min. Then, the solution was used for RAW264.7 cell culture and transfection. After another 48 h incubation, the cells were collected for flow cytometry analysis.

*In vivo* mRNA transfection

Female BALB/c mice were shaved 24 h before the injection. Nanoparticles-mCherry mRNA complex (400 μg nanoparticles premixed with 10 μg mCherry mRNA) or *in vivo*-jetPEI-mCherry mRNA complex (based on the manufacture’s protocol) were subcutaneously injected to the right flank of mice. The mCherry expression was visualized by IVIS Lumina X5 Optical Imager (PerkinElmer). Major organs and lymph nodes were dissected and collected for visualization by IVIS imaging system.

Statistical analysis

Statistical analysis was performed using multiple t test. *** shows p<0.0005, ** shows p<0.005, * shows p<0.05, *NS* (p>0.05) indicates no significant difference.


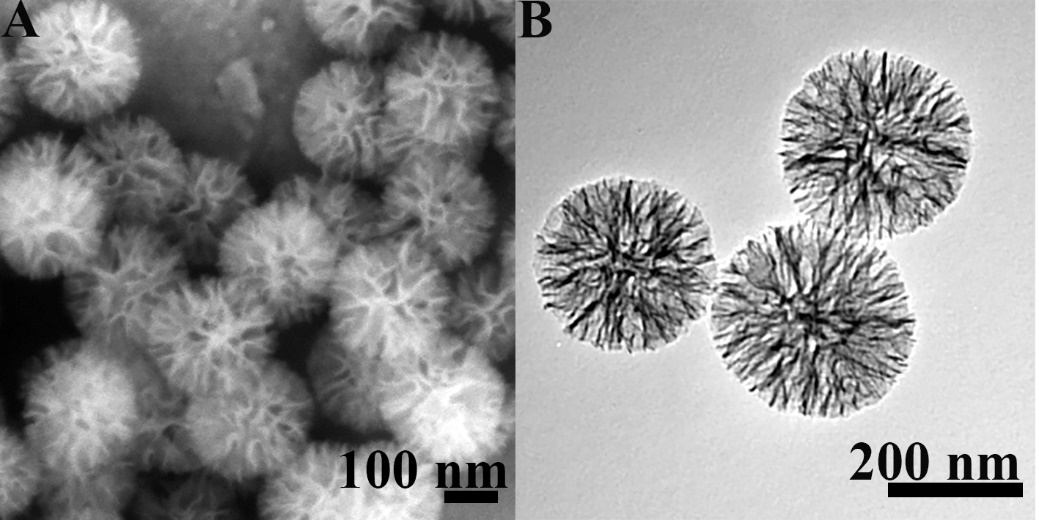


Figure S1. SEM (A) and TEM (B) images of DMONs.


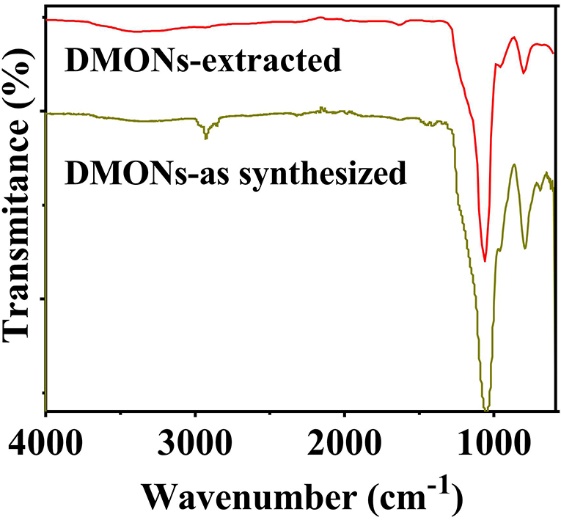


Figure S2. FTIR spectrum of DMONs before and after acid extraction.


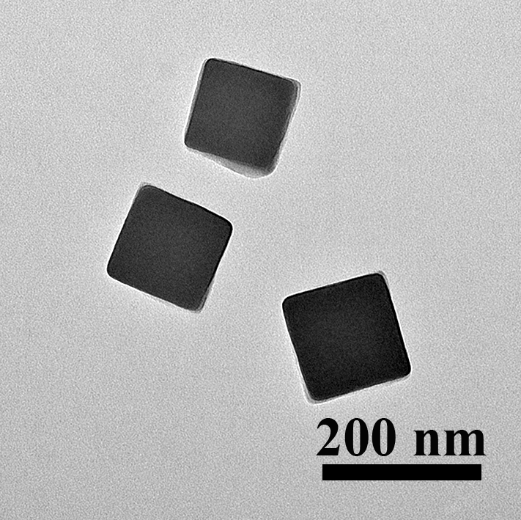


Figure S3. TEM image of ZIF-8 nanocube.


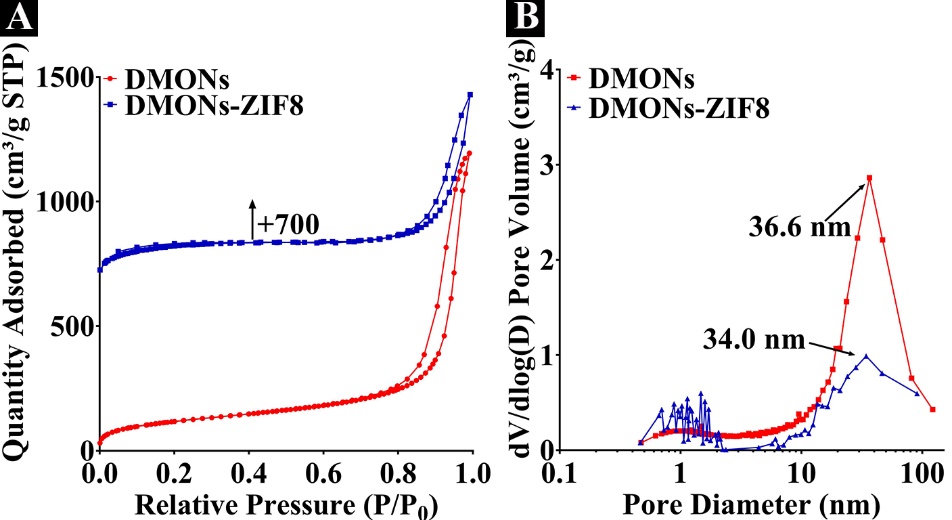


Figure S4. Nitrogen sorption adsorption-desorption isotherms and pore size distribution (B) of DMONs and DMONs-ZIF-8.

Table S1 Structural properties DMONs before and after ZIF-8 modification

| Sample name | S_BET_ (m^2^ g^-1^) | V_total_ (cm^3^/g) | Nitrogen (%) |
| --- | --- | --- | --- |
| DMONs | 405 | 1.85 | 0 |
| DMONs-ZIF-8 | 483 | 1.13 | 2.55 |


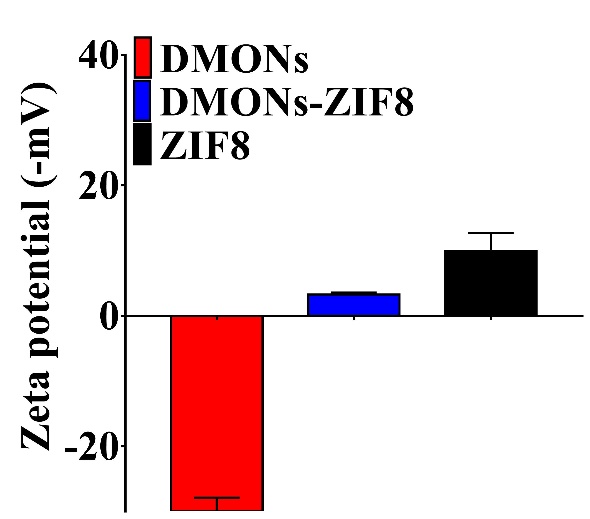


Figure S5. Zeta potential measurement (I) of DMONs, DMONs-ZIF-8, ZIF-8 nanocube and DMONs-PEI in water.


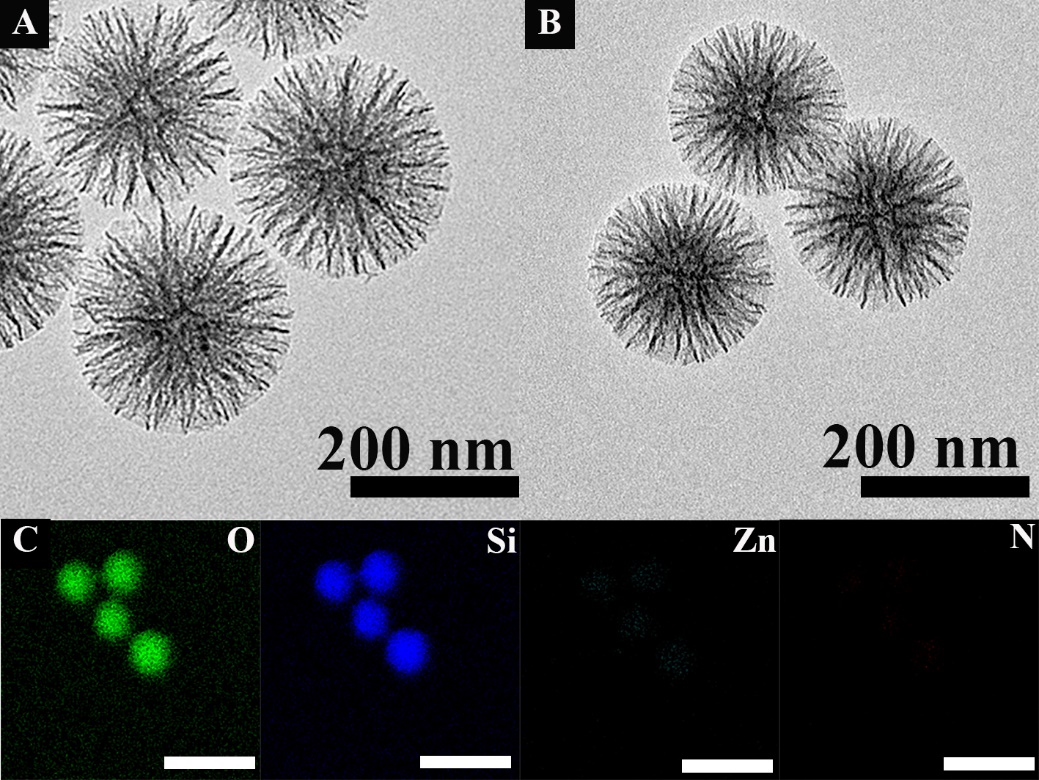


Figure S6. TEM images of DMSNs before (A) and after (B) ZIF-8 growth. EDX mapping (C) of DMSNs after ZIF-8 growth.


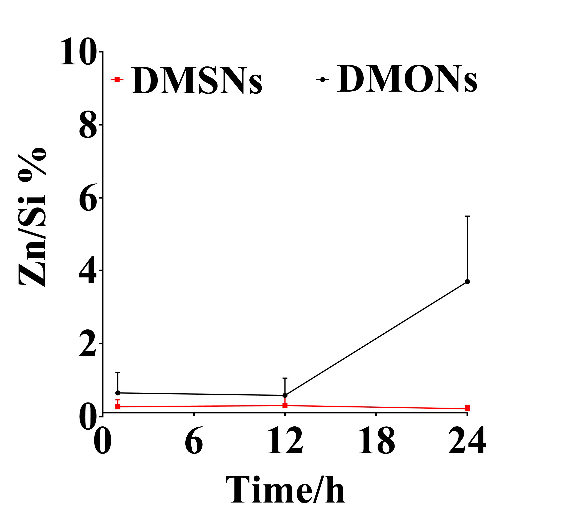


Figure S7 Time dependent Zn/Si% in sedimentation analysed by ICP-OES after mixing zinc nitrate with DMONs/DMSNs methanol solution


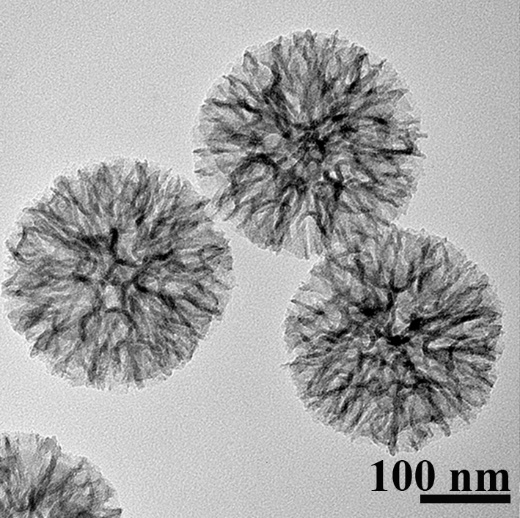


Figure S8 TEM image of DMONs-PEI.


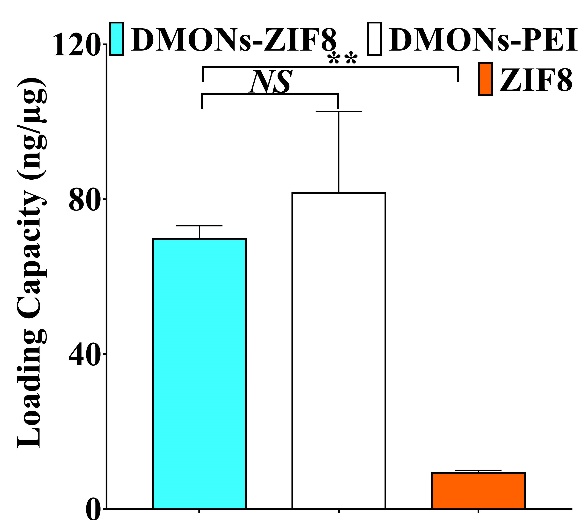


Figure S9. EGFP mRNA loading capacity in PBS (pH=7.4).


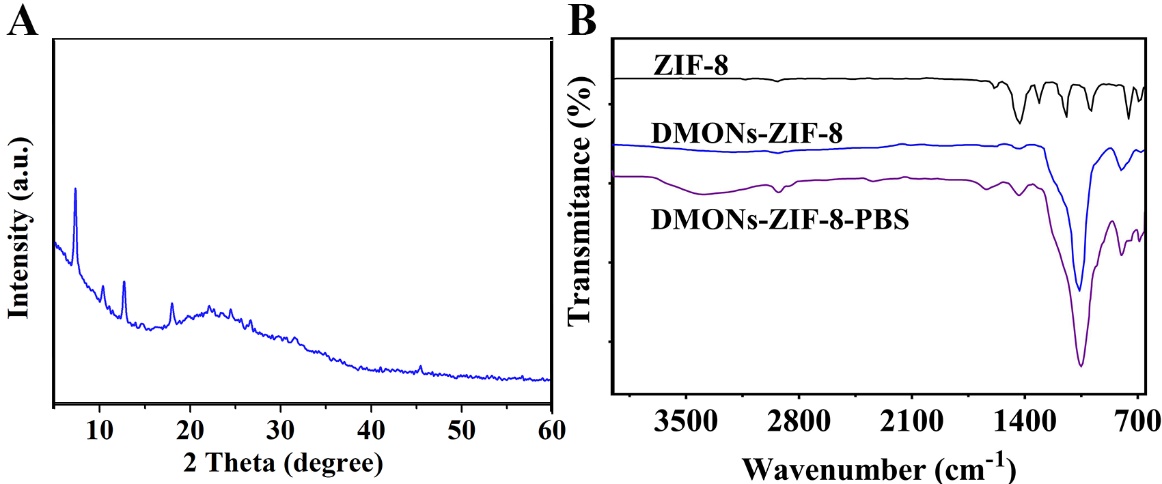


Figure S10. XRD pattern of DMONs-ZIF-8 after PBS treatment (A) and FTIR spectra of ZIF-8, DMONs-ZIF-8 before and after PBS treatment (B).


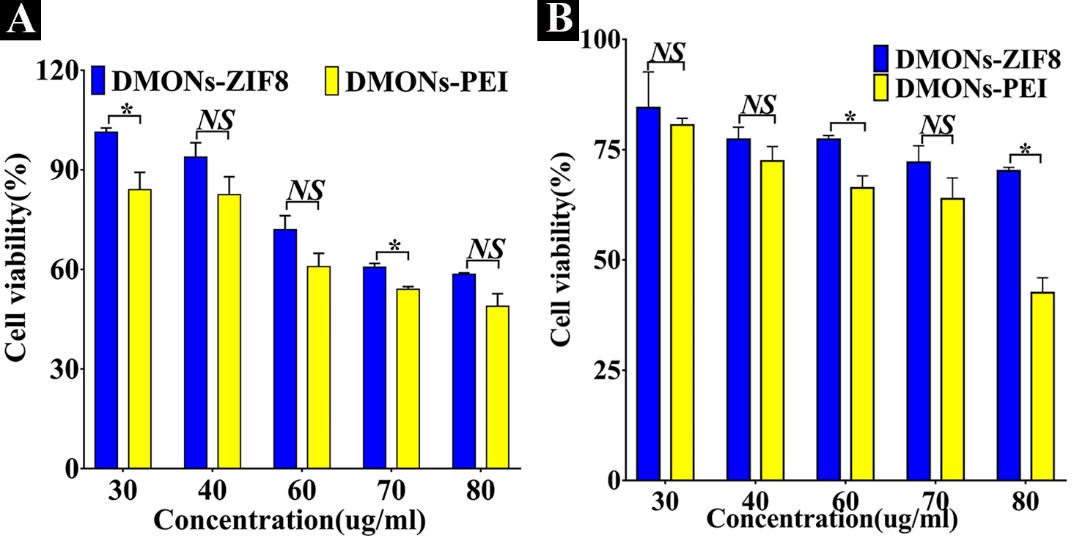


Figure S11. Cytotoxicity result of NPs incubated with A)RAW264.7 cells and B) HEK293T cells for 24 h. * shows p<0.05, *NS* (p>0.05) indicates no significant difference.


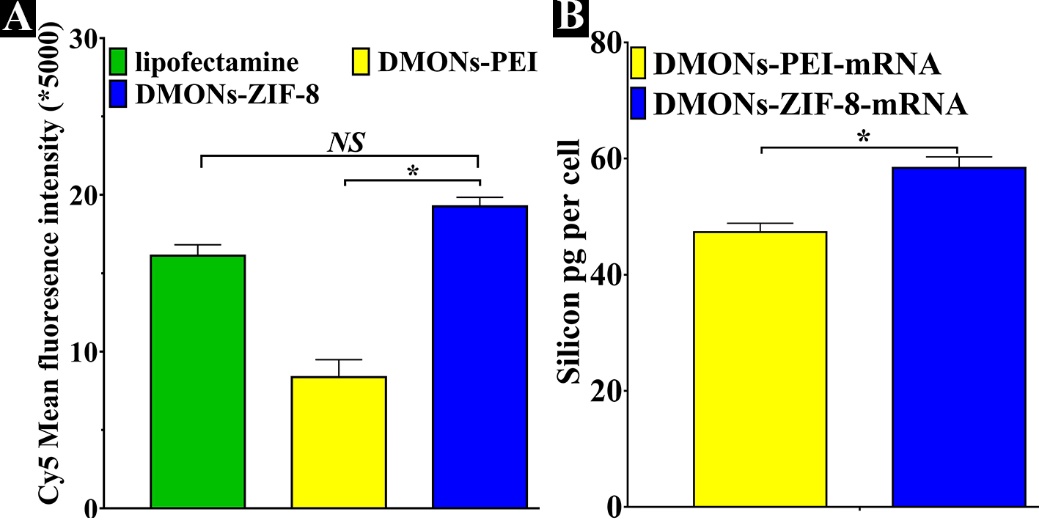


Figure S12. (A) The MFI of Cy5 measured by flow cytometry in RAW264.7 cells incubated with Cy5-mRNA / nanoparticles or lipofectamine formulations for 4 h. (B) The silicon content per cell determined by ICP analysis in RAW264.7 cells incubated with DMONs-PEI/ZIF-8-mRNA complex for 4 h.


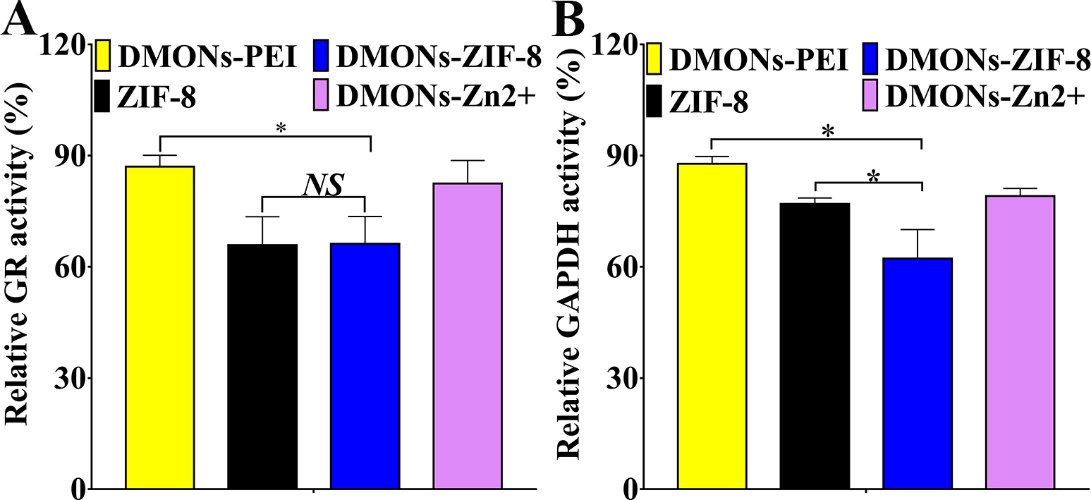


Figure S13. GR (A) and GAPDH (B) activity of RAW264.7 cells incubated with DMONs-PEI, ZIF-8, DMONs-ZIF-8 and mixture of DMONs and zinc nitrate for 24 h.


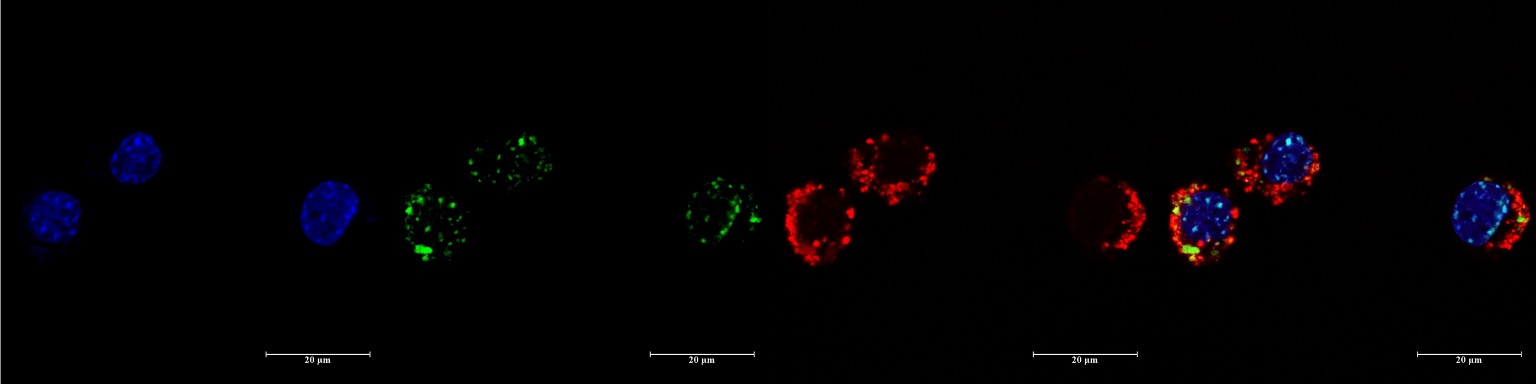


Figure S14. Confocal images (E) of DMONs-ZIF-8/Cy5-mRNA complex incubated with RAW264.7 for 8 h with mRNA concentration of 1 µg/ml, scale bar: 20 µm.


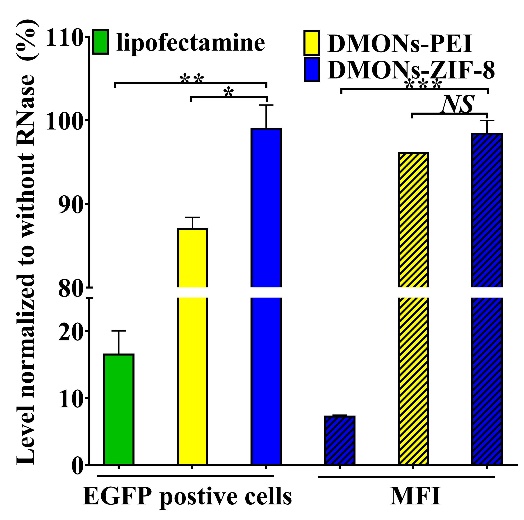
Figure S15. The relative level of EGFP positive cells or MFI after RNase treatment compared to the same group without treatment after 48 h transfection in RAW264.7 cells analyzed by flow cytometry.


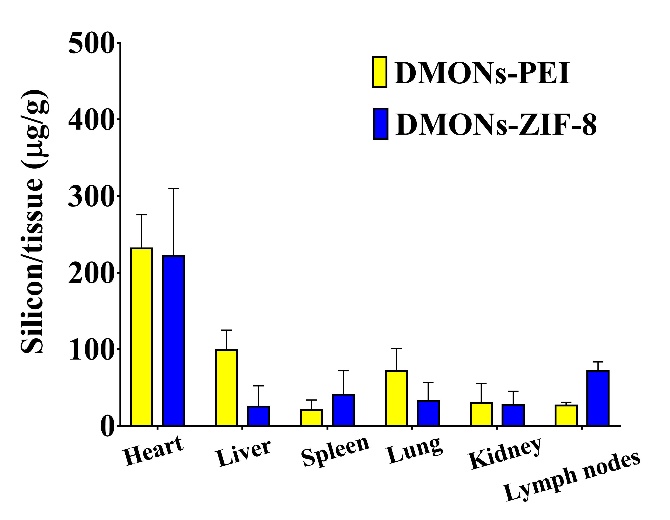


Figure S16. Silicon biodistribution in major organs quantified by ICP after 48 h injection of nanoparticle/mRNA formulations.


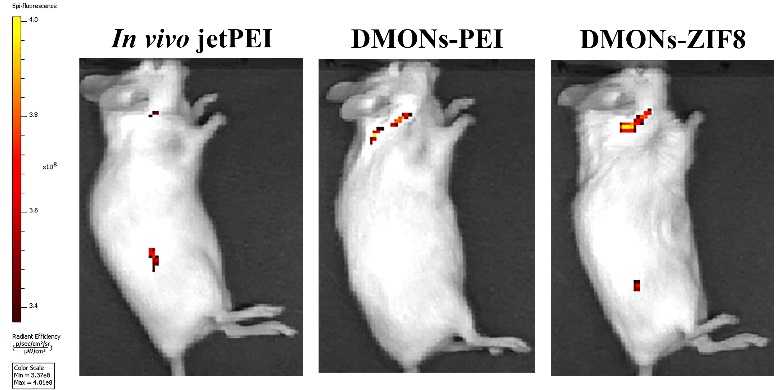


Figure S17. Fluorescence images of mCherry mRNA transfection in BALB/c mice after 17d.
